# Geographic isolation and environmental heterogeneity shape population genetic differentiation of a medicinal plant, amla (*Phyllanthus emblica* L.) in river valleys of Yunnan, China

**DOI:** 10.3389/fpls.2025.1648822

**Published:** 2025-09-10

**Authors:** Cai-yun Wang, Jia-cong Huang, Ming-yu Yin, Hong-jiang Hu, Yan-ping Yang, Jun-jie Guo, Jie Zeng

**Affiliations:** ^1^ Research Institute of Tropical Forestry, Chinese Academy of Forestry, Longdong, Guangzhou, Guangdong, China; ^2^ Forestry and Grassland Technique Promotion Station of Baoshan City, Baoshan, Yunnan, China

**Keywords:** *Phyllanthus emblica*, SSR, genetic diversity, environmental factors, geographic isolation

## Abstract

The diverse topographies of high mountains and deep river valleys in Yunnan, China create geographic and environmental barriers that promote intraspecific genetic differentiation. This study employed amla (*Phyllanthus emblica* L.) to reveal the effects of geographic and environmental isolation on genetic differentiation of plant species. We sampled 18 natural *P. emblica* populations from upstream to downstream in the Longchuan, Nu, Lancang, Yuanjiang and Jinsha Rivers valleys (three or four per valley) in 2017. Genetic diversity and structure of *P. emblica* were assessed across these populations using 16 SSR loci developed, and analyzed with GenAlEx, ATetra, and STRUCTURE software packages. Ecological niche modeling (MaxEnt) estimated its historical and contemporary potential geographic distribution patterns, and redundancy analysis (RDA) identified key environmental factors influencing genetic diversity. *P. emblica* exhibited high genetic diversity, primarily influenced by mean temperature of the warmest quarter (Bio10) and precipitation of the warmest quarter (Bio18). STRUCTURE analysis revealed a distinct division of these populations into western and eastern groups, closely aligned with the Tanaka-Kaiyong Line. Significant genetic differentiation existed between the western and eastern populations, and suggesting that long-term geographic isolation and environmental heterogeneity promote genetic differentiation and local adaptation of this species. MaxEnt modelling indicated a significant expansion in the potential habitat of *P. emblica* from the Last Glacial Maximum (LGM) to the present, likely due to climate warming. Our findings provide evidence for genetic differentiation in *P. emblica* driven by geographical and environmental isolation, and offer critical insights into developing effective conservation and utilization strategies for its genetic resources.

## 1 Introduction

Mountain uplift events and climatic oscillations during ancient geological periods jointly drive species divergence mechanisms intra- and interspecific levels, fundamentally shaping current biodiversity patterns (Hewitt, 2000; He and Jiang, 2014; Favre et al., 2015; Badgley et al., 2017). Genetic differentiation generally occurs in allopatric populations of a species due to geographic barriers and environmental isolation (Meng et al., 2017; Xing and Ree, 2017). For example, Wu et al. (2024), found that the *Morella nana* population on the Yunnan-Guizhou Plateau was divided into two groups, one in the eastern and the other in the western part of the Wumeng Mountains. This distribution pattern was likely related to the geographical isolation of the Wumeng Mountains and climate fluctuation during the last glacial maximum. Li et al. (2019), considered that mountain barriers enhanced the genetic differentiation of *Quercus chenii* populations through both geographic and environmental isolation in East China. Jinga and Ashley (2018), found that the Kalahari-Zimbabwe axis forms a strong genetic barrier between populations of *Afzelia quanzensis* in the northern and southern regions. Understanding the impact of geographic isolation and environmental heterogeneity on genetic diversity and population genetic structure of a species will help unravel its complex evolutionary history and its responses to climate change.

Yunnan is located in southwestern China, and its terrain is mainly characterized by a gradual descent from the northwest to the southeast, intersected by mountain ranges and deeply incised river valleys. The most notable rivers are the Nu, Lancang, Yuanjiang and Jinsha Rivers, along with their tributaries (Li and Zhao, 2007; Wang et al., 2017). The unique and intricate geomorphological landscape of this region, in synergy with its heterogeneous climatic regimes, has engendered a vast reservoir of genetic diversity through evolutionary adaptation processes. The genetic diversity and population genetic structure have been investigated in the river valleys of this region for some critically endangered and extremely small population plant species (Jia et al., 2016; Tang et al., 2020) as well as for a limited number of populations for widely distributed species (Li and Zhao, 2007; Liu et al., 2020).

Amla (*Phyllanthus emblica* L.; syn. *Emblica officinalis* Gaertner), a medicinal plant belonging to the *Phyllanthaceae* family, is widely distributed across tropical and warm subtropical regions in China, Southeast Asia, and South Asia. In China, it primarily occurs in Yunnan, Guangxi, Guangdong, Hainan, Fujian, Guizhou and Sichuan provinces (Ma et al., 2024). *P. emblica* is a deciduous tree characterized by thin, light grey bark that exfoliates in small irregular flakes, small light green leaves densely set along the branchlets, and globose fruits with six vertical furrows (Saini et al., 2022). As an economically significant plant, amla plays a crucial role in regional agriculture and forestry. Its fruits are rich in compounds including vitamin C, pentagalloylglucose, and gallic acid (Krishnaiah et al., 2009; Ma et al., 2024). Modern pharmacological studies have shown that *P. emblica* possesses various pharmacological activities, such as antioxidant, anti-tumor, hypoglycemic, and sore throat-relieving effects (Chaphalkar et al., 2017; Variya et al., 2016). This species exhibits tolerance to barren soils and possesses strong soil conservation capacities, contributing to its ecological value. Its pollen dispersal, primarily mediated by bees and wind, facilitates long-distance gene flow between populations (Nakanishi et al., 2020). Yin et al. (2018) assessed phenotypic diversity in fruits, seeds and offspring seedlings across nine natural *P. emblica* populations in the Nu and Longchuan River valleys of western Yunnan, finding significant correlations between most seed and seedling traits and geographical and environmental factors. However, the influence of geographic distance and environmental variables on the genetic diversity and population genetic structure of *P. emblica* within these river valleys of Yunnan remain poorly understood.

In this study, we developed polymorphic SSR markers and used them to analyze the genetic diversity and structure across 18 natural *P. emblica* populations from five river valleys in Yunnan. We hypothesized that complex topography drives genetic differentiation through two primary mechanisms: (1) geographic isolation imposed by mountain barriers promotes population divergence, and (2) environmental heterogeneity in valleys generates adaptive genetic variation. Our objectives were: (i) to investigate effects of geographic isolation and environmental heterogeneity on genetic diversity and population genetic structure; and (ii) to reconstruct past and present geographic distribution using ecological niche modelling. This approach quantifies correlation between species occurrence and environmental variables to describe ecological niches and habitat suitability (Guisan and Zimmermann, 2000; Gao et al., 2022). The findings will not only advance our understanding of how geographic and environmental isolation shape genetic differentiation in complex terrains, but also provide a foundation for developing conservation and utilization strategies for this species.

## 2 Materials and methods

### 2.1 Plant materials and DNA extraction

**Figure 1:**
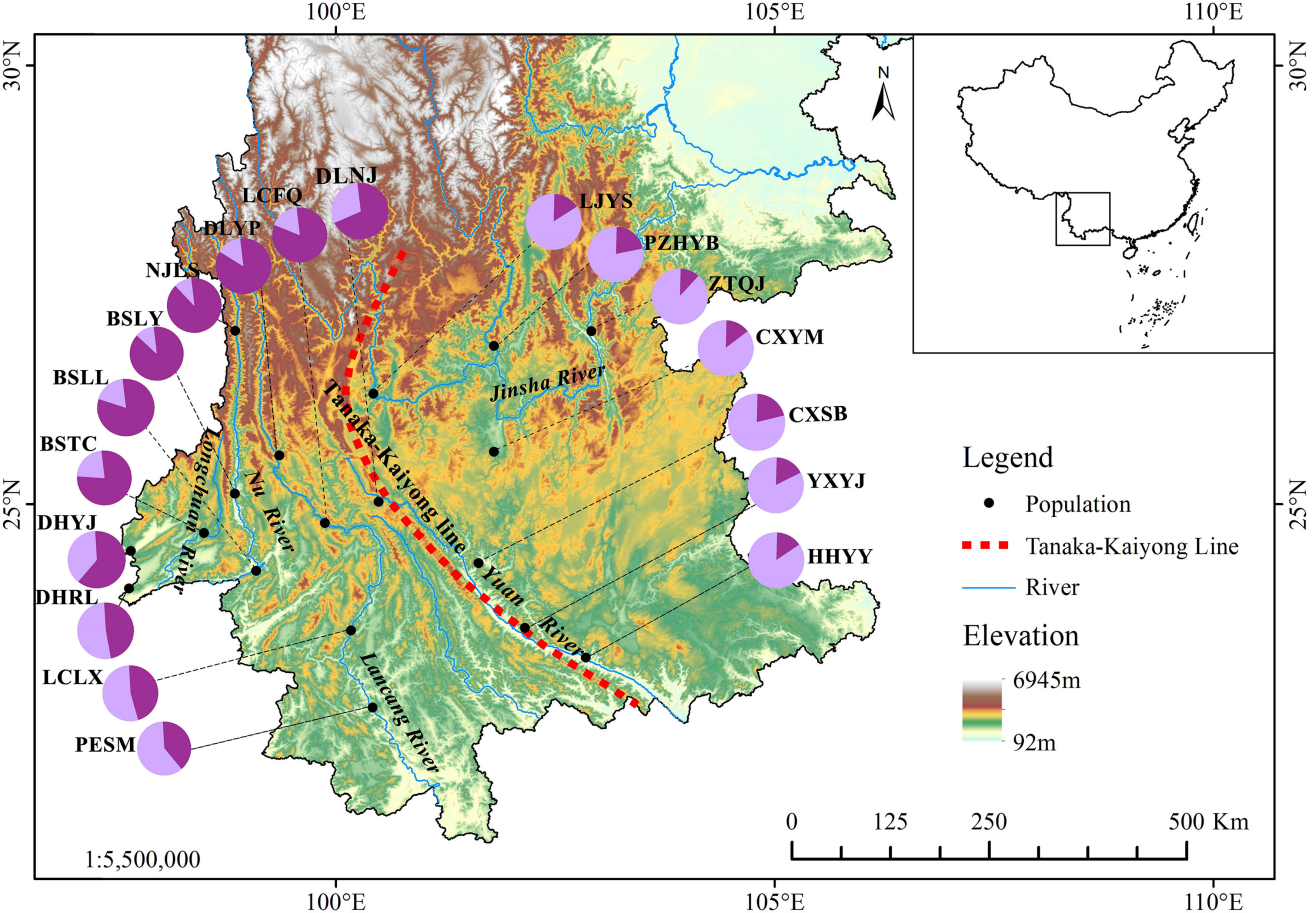
Localities of 18 natural *Phyllanthus emblica* populations in the valleys of five rivers of Yunnan, China. The pie chart shows the proportions of two genetic groups assigned to each population based on STRUCTURE analysis. Population information is provided in **Table 1**. The delineation of the Tanaka-Kaiyong Line is based on Fan et al. (2013).

The sampling sites were primarily located in the valley areas of five rivers, including the Longchuan, Nu, Lancang, Yuanjiang and Jinsha Rivers, in Yunnan and Sichuan Provinces (**Figure 1**). In each river valley, three or four natural populations of *P. emblica* were sampled from upstream to downstream in 2017. More than 30 individuals were sampled from each population, with a minimum distance of 50 m between them (**Table 1**). In total, 564 leaf samples were collected from 18 natural *P. emblica* populations in the region. The sampled leaves were dried using silica gel, and their total DNAs were extracted using the modified CTAB method (Zeng et al., 2002).

**Table 1:** General information of 18 natural *Phyllanthus emblica* populations from the valley areas of five rivers.

Valleys	Population code	Location (county, province)	Latitude	Longitude	Altitude range (m)	Sample size
Longchuan River	BSTC	Tengchong,Yunnan	24.67572	98.49651	1388-1455	30
	DHYJ	Yingjiang,Yunnan	24.47061	97.66352	842-941	30
	DHRL	Ruili,Yunnan	24.04469	97.64129	870-1011	33
Nu River	NJLS	Lushui,Yunnan	26.98148	98.85150	887-1071	31
	BSLY	Longyang,Yunnan	25.12343	98.84662	798-988	30
	BSLL	Longling,Yunnan	24.24299	99.09122	779-945	30
Lancang River	DLYP	Yongping,Yunnan	25.55952	99.35273	1272-1306	31
	LCFQ	Fengqing,Yunnan	24.78641	99.87317	1292-1415	30
	LCLX	Linxiang,Yunnan	23.56376	100.17170	867-992	33
	PESM	Simao,Yunnan	22.68325	100.41681	1208-1238	30
Yuan River	DLNJ	Nanjian,Yunnan	25.03004	100.48739	1430-1513	33
	CXSB	Shuangbai,Yunnan	24.33127	101.62621	648-681	32
	YXYJ	Yuanjiang,Yunnan	23.55208	102.10265	717-815	32
	HHYY	Yuanyang,Yunnan	23.25591	102.84756	425-514	32
Jinsha River	LJYS	Yongsheng,Yunnan	26.26231	100.42617	1277-1354	32
	PZHYB	Yanbian,Sichuan	26.80581	101.80011	1026-1200	32
	CXYM	Yuanmou,Yunnan	25.60337	101.80055	1414-1638	31
	ZTQJ	Qiaojia,Yunnan	26.97432	102.91080	1021-1182	32

### 2.2 SSR marker development

Genomic DNA from a single sample of *P. emblica* was broken into fragments of 300–500 bp in length. The paired-end sequencing library was constructed with NEXTflex Rapid DNA-Seq Kit (Bioo Scientific Corp., Austin, USA) following the manufacturer’s instructions. According to the sequencing methodology described in Guo et al. (2017). The sequencing data have been deposited in GeneBank. Prior to the genome assembly, quality control was conducted on the raw data by discarding reads containing more than 10% of bases with a quality score (Q) below 20, reads containing more than 10% ambiguous sequences, and those with adaptor contamination. *De novo* genome assembly was then performed using SPAdes 3.9.0 software (Bankevich et al., 2012) with the default settings.

SSRs were detected in the assembled genome using the MISA tool (Labbé et al., 2011), targeting motifs from di- to hexa-nucleotides. The minimum repeat numbers were set as follows: five for dinucleotide, four for trinucleotide, and three for tetranucleotide, pentanucleotide and hexanucleotide. The maximal number of bases interrupting two SSR loci was 20 in a compound microsatellite. One hundred and ten primers were designed using Primer 3 v4.1.0 (Untergasser et al., 2012) (http://fokker.wi.mit.edu/primer3/) with default parameters.

The PCR reaction mixture (10 μL) contained 50 ng of DNA template, 150 μM dNTPs, 2.0 μM MgCl_2_, 0.5 μM forward and reverse primers, 1× PCR buffer (Tiangen Biotech Ltd., Beijing, China), and 0.04 U/μL of Taq DNA polymerase (Tiangen Biotech Ltd). PCR was performed on an Applied Biosystems Veriti thermal cycler (Applied Biosystems, Waltham, Massachusetts, USA) with the following program: 94°C for 4min; 94°C for 30 s, annealing temperature (*Ta*) for 30 s, 72°C for 30 s (31 cycles); and 72°C for 10 min. The optimized *Ta* were determined as 60°C through a PCR trial with temperature gradient. The PCR products were detected by electrophoresis on 1% agarose gel. The PCR products were analyzed using ABI 3730XL (Applied Biosystems, USA) with GeneScan 500 LIZ Size Standard (Applied Biosystems, USA). Genotyping was performed using GeneMarker v2.2 software (Hulce et al., 2011).

### 2.3 Genetic diversity and genetic structure analysis

The SSR primers developed were further used to conduct SSR analysis for all samples using the methods outlined above. Since *P. emblica* is of complex ploidy (Rahman et al., 2021), the genotype data were converted into binary data (0, 1) using the poppr package in R (Kamvar et al., 2014; López-González et al., 2021; Thapa et al., 2021). The number of alleles (*N*a), the observed heterozygosity (*Ho*) and private allele (*Ap*) were calculated for each *P. emblica* population using Excel 2010 software. The expected heterozygosity (*He*) and the Shannon-Weiner diversity index (*I*) were estimated with ATetra v1.2 software (Markwith et al., 2006; Van-Puyvelde et al., 2010). *H*e and *I* were calculated with the formulae $He=1-\sum p_{i}^{2}$ and $I=-\sum p_{i}^{2}{\text{log}}_{2}p_{i}$ , where *P_i_* is the frequency of the *i*th allele at a given locus within a population. Analysis of molecular variation (AMOVA), principal component analysis (PCoA), and calculation of Nei’s genetic distance were performed using GenAlEx v6.51 software (Peakall and Smouse, 2006). Genetic differentiation values (PhiPT) were calculated for each pair of river valleys or populations on the basis of AMOVA. An unweighted pair group method with arithmetic mean (UPGMA) dendrogram was constructed based on Nei’s genetic distance using MEGA v7.0 (Kumar et al., 2016). The genetic structure of *P. emblica* was examined using STRUCTURE v2.3.4 software (Pritchard et al., 2000). The number of clusters (*K*) was tested from one to 18, with 10 runs for each K. The burn-in length was set to 100,000, with Markov Chain Monte Carlo iterations of 100,000. The optimum *K* value was determined based on the highest delta *K* value, using STRUCTURE Harvester on-line (Earl and vonHoldt, 2012).

Altitude and six climatic variables (**Supplementary Table S1**) were selected from 20 environmental factors (See subsection 2.4 Ecological niche modelling) to avoid multicollinearity. This was achieved by calculating Pearson’s correlation coefficients and retaining only one variable per correlated group (|r|≥0.8) (Zhu et al., 2025). These seven selected variables were subsequently used in further analyses. The Mantel and partial Mantel tests were conducted 9999 times using the vegan package in R software to assess correlation between genetic differentiation (Bray-Curtis distance) and geographic distance or these selected environmental variables (Euclidean distance). Redundancy analysis (RDA) was employed to identify whether these selected environmental factors significantly influencing genetic diversity or not, using Canoco v5.0 software (Morris, 2015).

### 2.4 Ecological niche modelling

Data for all but of 18 populations in Yunnan were mainly obtained from online species information resources after species identification and distribution calibration, including the Chinese Virtual Herbarium (https://www.cvh.ac.cn/), the Global Biodiversity Information Facility (http://www.gbif.org) and related references. Coordinates were picked through the Baidu Maps coordinate system. A total of 97 distribution points of *P. emblica* were obtained for model prediction. Data of 19 climatic variables and one topographic factor (altitude) were sourced from the Global Climate Database (http://www.worldclim.org/) for three periods (Last Glacial Maximum; Middle Holocene; Present 1970-2000). MaxEnt v3.4.4 software was used to import geographic distribution data and the environment variable layer into the model, and the analysis was performed using the jackknife method. The prediction maps from the MaxEnt model were imported into ArcGIS10.1, where the viable range was divided into four levels using the reclassification tool.

## 3 Results

### 3.1 SSR marker development

A total of 346,029 contig sequences were obtained in the *P. emblica* genome, with an average length of 193 bp. Among all contigs examined, 8511 contained SSR loci, of which 5965 could be used to design PCR primers, with the expected product sizes ranging from 100 to 280 bp. Of the 110 pairs of SSR primers designed, 58 could generate polymorphic loci. Subsequently, 16 pairs of SSR primers with good amplification performance were selected to assess the genetic diversity and structure across 18 natural *P. emblica* populations (**Table 2**).

**Table 2:** Characterization of 16 microsatellite (SSR) loci for *Phyllanthus emblica*.

Locus	Primer sequences (5’-3’)	Repeat motif	Allele size range (bp)	*T_a_* (°C)	*Na*	*Ap*	*Ho*	*He*	*I*	GenBank Accession No.
PEH002	F:TGCCACCACTCAACAGTTAGTT	(GTAT)_7_	209–285	60	19	2	0.761	0.801	1.971	MK017818
	R:GGAATACTTGTGCTCACCCTCA									
PEH005	F:TTCCCTACACACCACTACTCCA	(AGCC)_4_	261–285	60	7	2	0.857	0.602	1.018	MK017800
	R:GCAGAGCTTCAAAGAAAGGTGG									
PEH007	F:CCTCTTGTTGTTGCTGAGAAGC	(TCAA)_4_	117–137	60	6	0	0.752	0.514	0.779	MK017799
	R:CTCCAGACAGAACAGAAGGTGT									
PEH012	F:GACAGCCTCAACCCTTTTCAAC	(CCT)_7_	284–302	60	7	0	0.603	0.592	1.101	MK017801
	R:TGTTAATTGTAATCCCACGCGC									
PEH031	F:TTTGCCAAATACTGATCCCCGA	(TG)_10_	110–164	60	25	3	0.965	0.791	1.887	MK017802
	R:TGCCTTTACACTGCACCATTCT									
PEH032	F:TCCTGCTTCTGAGTTCCATTCA	(AG)_10_	147–201	60	23	1	0.848	0.818	1.985	MK017803
	R:CTAGCACAATGGCAAGGCATAC									
PEH037	F:AGACAGGTTTATCACATCCACTGT	(AT)_9_	123–139	60	9	3	0.430	0.632	1.140	MK017804
	R:ACAGCCATGTAGTTAACCGTGT									
PEH044	F:TGGTTGGTTCACTTTACACCCA	(CT)_8_	188–200	60	6	0	0.759	0.597	1.038	MKO17805
	R:GTTTTTCCCTGCCACCAACTAC									
PEH047	F:ATTGTTTACGCACAGTGGTTGG	(TG)_8_	143–155	60	6	1	0.948	0.579	1.068	MK017806
	R:GACAGGTATTATGGAGGTGGCT									
PEH051	F:AGTGTGAGTGTGTTGTCCTGTT	(TG)_7_	141–151	60	6	1	0.983	0.567	0.950	MKO17807
	R:AACACTAACACTCCCCCTTTCC									
PEH055	F:CATTGATGTGGGGACAAGCTTG	(TG)_7_	157–177	60	11	1	0.907	0.738	1.533	MK017808
	R:GAACGCATTCAGCCTGTGTTTT									
PEH056	F:AATACCCGAACCAACGATTCCA	(CT)_7_	240–274	60	14	4	0.857	0.669	1.291	MK017809
	R:GGCTTTGCCGTTTTAGTTCACT									
PEH075	F:ACAGCACAACACACTTCTCTGA	(TC)_8_	196–222	60	14	0	0.909	0.702	1.460	MK017810
	R:CCCGGTGTCATATCAGTCGAAT									
PEH079	F:TCACCCATAGCCAAATACCACA	(GT)_6_	115–131	60	7	3	0.811	0.616	1.105	MK017811
	R:AAGAAGCTGTCTTTTCGCAAGC									
PEH096	F:CGTCCATTTCAAAACGTCTCCT	(TC)_10_	154–200	60	23	2	0.996	0.899	2.462	MK017813
	R:AATGACCTCGAAGTTGGTGGAA									
PEH098	F:AGGGGTTTTGAGGCAGTAATGT	(AG)_9_	275–293	60	10	1	0.315	0.526	1.046	MK017814
	R:CACTTTTGGAAACGTGATTCTGC									

*Ta*, annealing temperature; *Na*, number of alleles; *AP*, number of private alleles; *Ho*, observed heterozygosity; *He*, expected heterozygosity; *I*, Shannon-Weiner diversity index.

### 3.2 Genetic diversity

A total of 193 alleles were detected at 16 SSR loci in 564 individuals from 18 populations of *P. emblica* across five river valleys (**Table 2**). The number of alleles (*Na*) per locus varied from six at loci PEH07, PEH44, PEH47 and PEH51 to 25 at locus PEH31 with overall mean of 12.063. The expected heterozygosity (*He*) and the Shannon-Weiner diversity index (*I*) ranged from 0.514 and 0.779 at locus PEH07 to 0.899 and 2.462 at locus PEH96, with means of 0.665 and 1.365, respectively. The number of private alleles (*Ap*) was high up to four at locus PEH56, while none was detected at loci PEH07, PEH12, PEH44 and PEH75 (**Table 2**).

*Na* of each population ranged from 6.125 in CXYM to 8.125 in LCLX, averaging 6.990. The *He* varied from 0.632 in LJYS to 0.701 in DLYP, with mean of 0.666. *I* ranged from 1.246 in LJYS to 1.476 in BSTC, averaging 1.367. LJYS exhibited the lowest level of genetic diversity. Populations LCLX and DLNJ had the most private alleles (three) among 18 populations, while none was detected in populations NJLS, BSLY, YXYJ, HHYY and CXYM (**Table 3**).

**Table 3:** Genetic diversity of 18 natural *Phyllanthus emblica* populations in five river valleys.

Valleys	Populations	*Na*	*Ap*	*Ho*	*He*	*I*
Longchuan River	BSTC	7.813	2	0.821	0.696	1.476
	DHRL	7.625	2	0.780	0.668	1.398
	DHYJ	7.063	2	0.776	0.640	1.327
	Mean	7.500	/	0.792	0.668	1.400
	Range	0.750	/	0.045	0.056	0.149
Nu River	NJLS	6.438	0	0.824	0.688	1.398
	BSLY	6.938	0	0.839	0.698	1.443
	BSLL	7.000	2	0.833	0.682	1.408
	Mean	6.792	/	0.832	0.689	1.416
	Range	0.562	/	0.015	0.016	0.045
Lancang River	DLYP	6.813	1	0.804	0.701	1.447
	LCFQ	7.125	1	0.776	0.676	1.404
	LCLX	8.125	3	0.738	0.648	1.362
	PESM	7.563	1	0.794	0.678	1.415
	Mean	7.407	/	0.778	0.676	1.407
	Range	1.312	/	0.066	0.053	0.085
Yuan River	DLNJ	7.313	3	0.845	0.682	1.403
	CXSB	7.250	1	0.738	0.645	1.323
	YXYJ	6.938	0	0.776	0.656	1.336
	HHYY	6.188	0	0.771	0.642	1.280
	Mean	6.922	/	0.783	0.656	1.336
	Range	1.125	/	0.107	0.040	0.123
Jinsha River	LJYS	6.188	2	0.740	0.632	1.246
	PZHYB	6.813	2	0.787	0.656	1.339
	CXYM	6.125	0	0.808	0.649	1.290
	ZTQJ	6.500	2	0.778	0.650	1.308
	Mean	6.407	/	0.778	0.647	1.296
	Range	0.688	/	0.068	0.024	0.093
Among-populations means		6.990	/	0.790	0.666	1.367
Species-level values		12.063	24	0.794	0.665	1.365

*Na*, mean number of alleles per locus; *Ap*, number of private alleles; *Ho*, observed heterozygosity; *He*, expected heterozygosity; *I*, Shannon-Weiner diversity index. Population information is shown in **Table 1**.

Among the five river valleys, the Jinsha River valley exhibited the lowest mean *Na* (6.406), *Ho* (0.778), *He* (0.647), and *I* (1.296), while the Longchuan River valley showed the highest mean *Na* (7.500), and the Nu River valley did the largest mean *Ho* (0.832), *He* (0.689) and *I* (1.416). Only two private alleles were detected in the Nu River, whereas six private alleles in the Longchuan, Lancang and Jinsha River valleys (**Table 3**). The populations in Nu River valley exhibited the narrowest ranges for *Na*, *Ho*, *He* and *I*, while the widest ranges for *Na* in the Lancang River valley, for *Ho* in the Yuan River valley, and for *He* and *I* in the Longchuan River valley.

From upper stream to downstream in each river valley, *Na* demonstrated a downward trend in the Longchuan and Yuan River valleys, an upward trend in the Nu River, and no clear pattern in the Langcang and Jinsha River valleys. *Ho*, *He* and *I* showed decreasing trend in the Longchuan, Lancang and Yuan River valleys, remained almost stable in the Nu River valley, and did not show any clear pattern in the Jinsha River valley (**Table 3**).

### 3.3 Population genetic structure and differentiation

**Figure 2:**
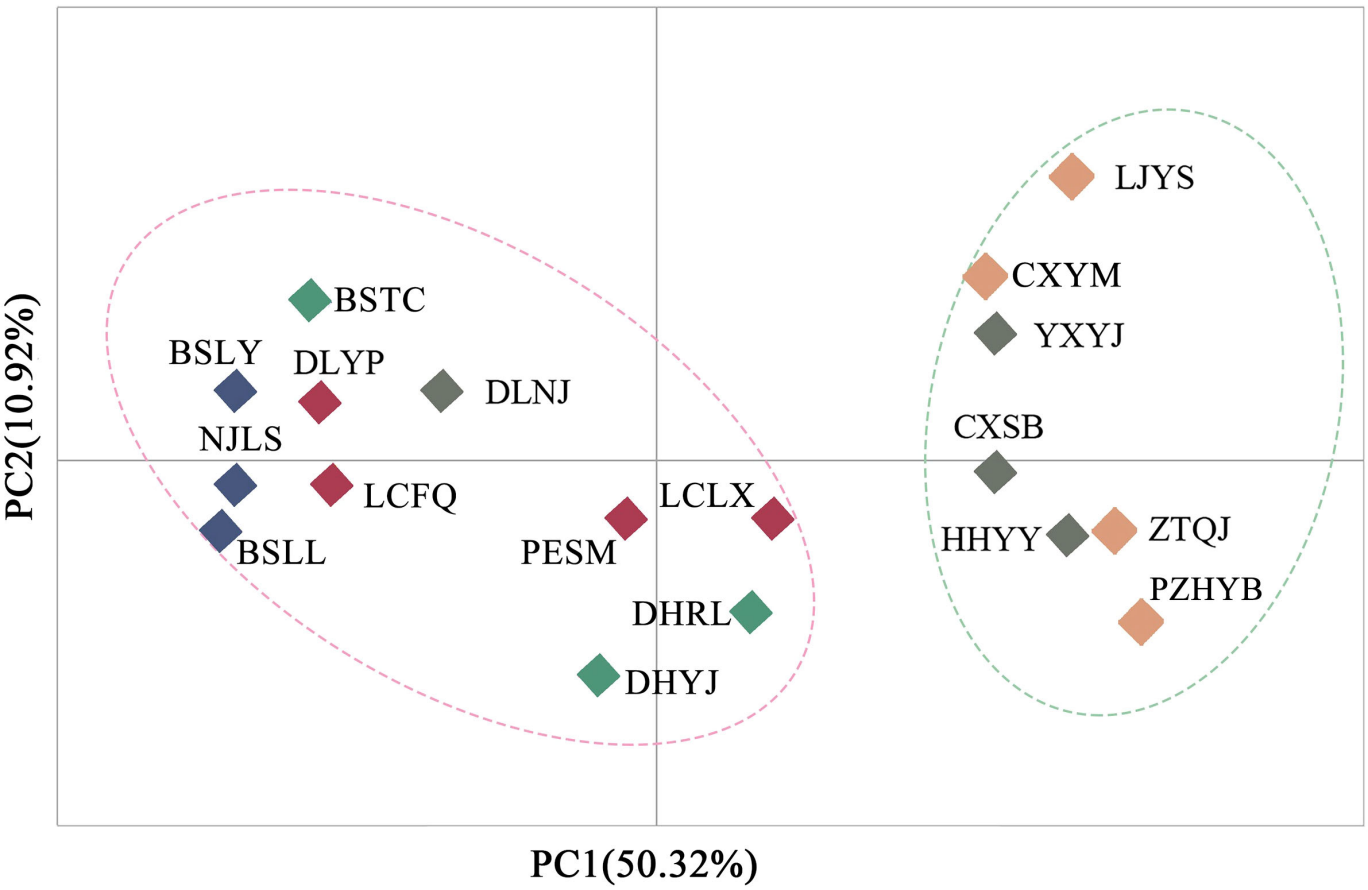
Principal component analysis (PCoA) for 18 natural *Phyllanthus emblica* populations based on genetic distance. Population information is shown in **Table 1**.

**Figure 3:**
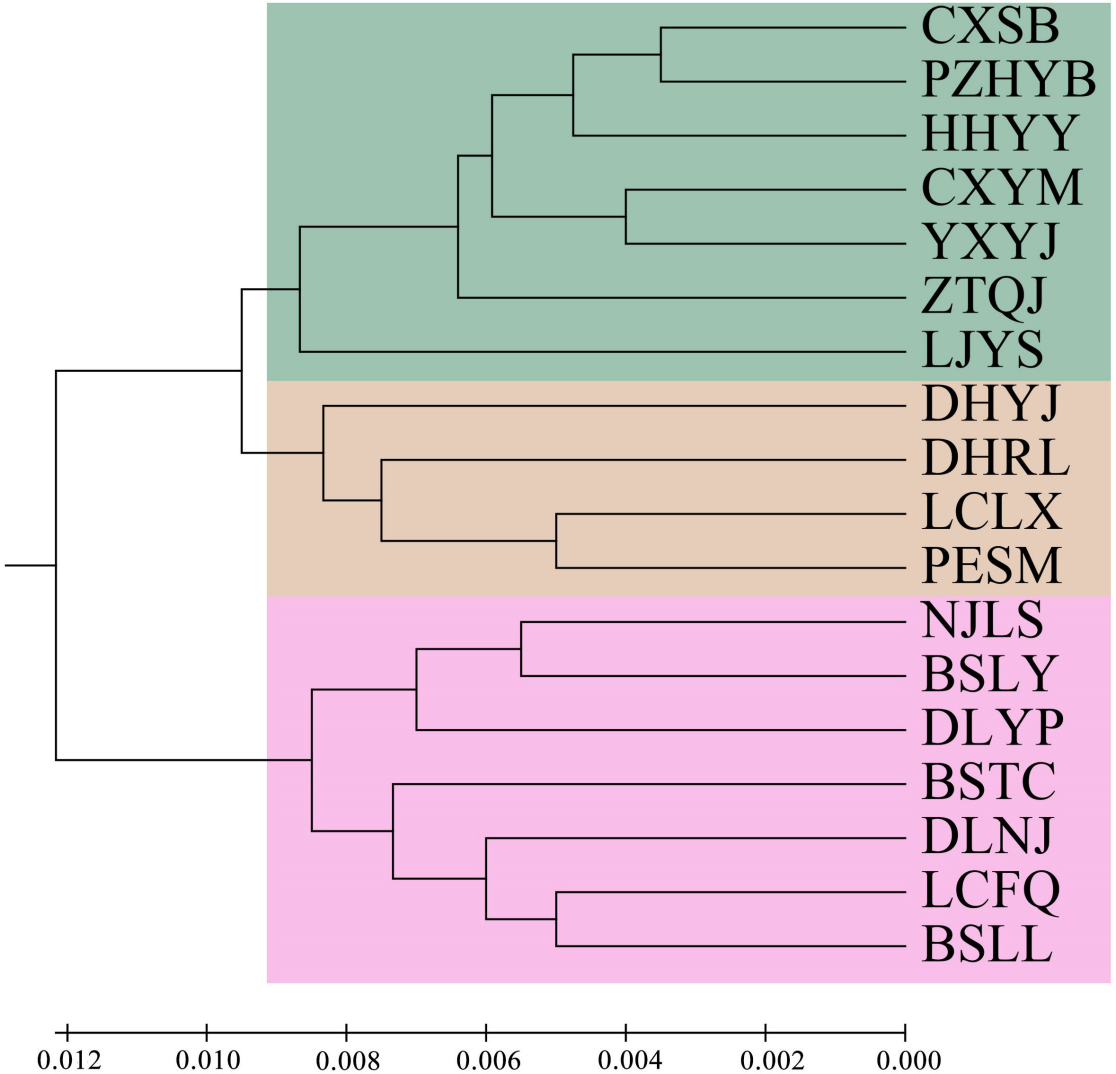
UPGMA dendrogram of 18 natural *Phyllanthus emblica* populations based on Nei’s genetic distances. Population information is shown in **Table 1**.

**Figure 4:**
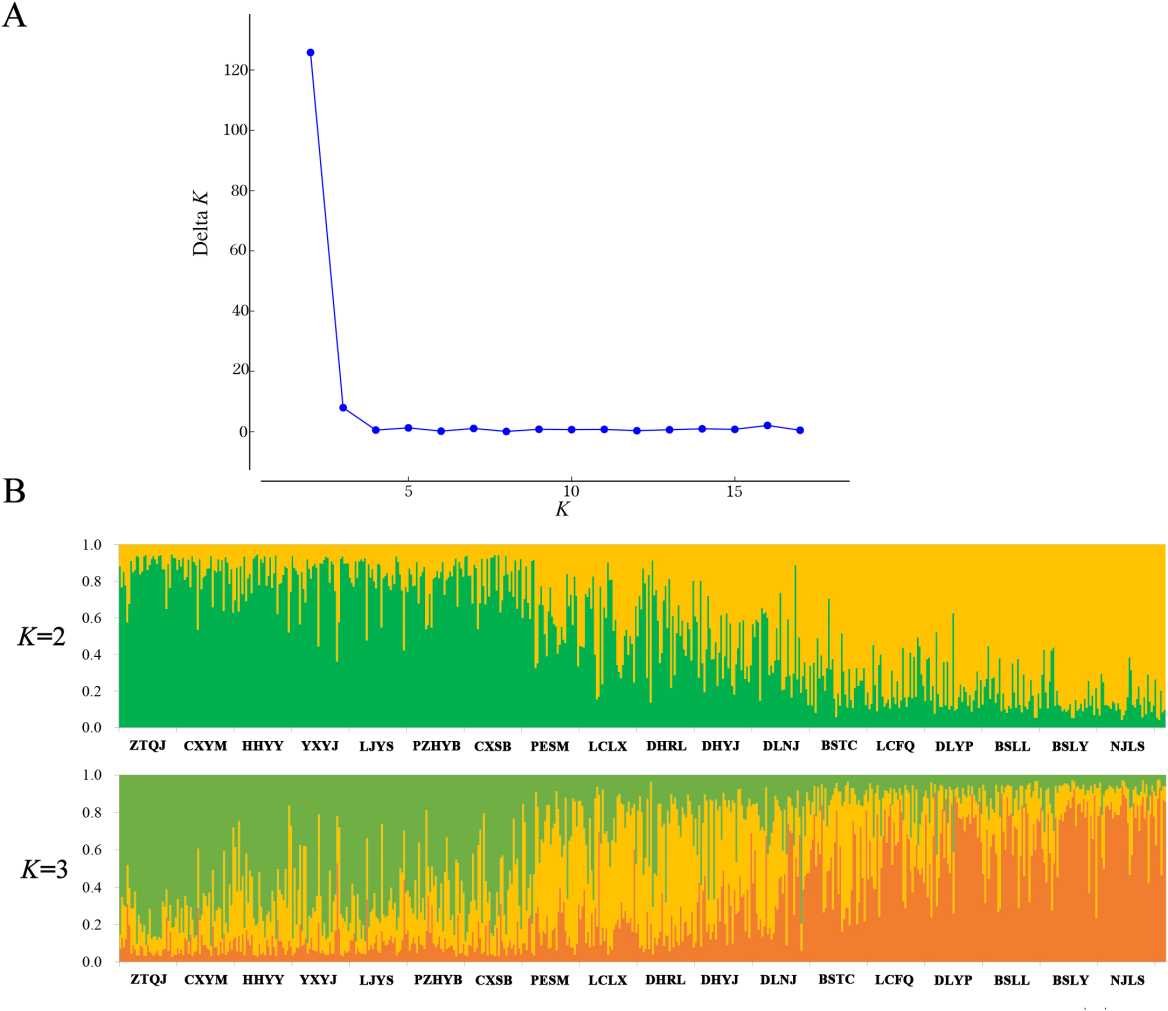
Genetic structure for 18 natural *Phyllanthus emblica* populations. **(A)** relationship between group number (*K*) of *Phyllanthus emblica* and estimated delta *K* revealed with STRUCTURE analysis. **(B)** the genetic structure plot of natural *Phyllanthus emblica* populations on the basis of STRUCTURE analysis. Population information is shown in **Table 1**.

Principal component analysis (PCoA) indicated that PCoA1 and PCoA2 explained 50.32% and 10.92% of the overall genetic variation, respectively (**Figure 2**). The 18 populations could be divided into two clusters. Cluster I included the populations from the Longchuan (BSTC, DHYJ, DHRL), Nu (NJLS, BSLY, BSLL), Lancang (DLYP, LCFQ, LCLX, PESM) and Yuan River valleys (DLNJ); Cluster II contained the populations from Yuan (CXSB, YXYJ, HHYY) and Jinsha River valleys (LJYS, CXYM, PZH, ZTQJ). Both clusters were just located on the western and eastern sides of the Tanaka-Kaiyong Line (see details in section Discussion). The unweighted pair group method with arithmetic mean (UPGMA) dendrogram resolved three clusters at a distance threshold of 0.009, with the western populations divided into two clusters, different from the PCoA grouping pattern (**Figure 3**). The STRUCTURE analysis identified *K* = 2 as optimal (**Figure 4A**), dividing 18 populations into two groups (**Figure 4B**) that aligned with PCoA clustering.

Analysis of molecular variation (AMOVA) revealed significant genetic variation (*p*<0.001) across three hierarchical levels: among river valleys (2.32%), among populations within river valleys (4.32%), and within populations (93.36%). The Longchuan River valley exhibited the highest (5.69%) while the Nu River valley the lowest (2.65%) genetic variations among populations (**Supplementary Table S2**). Taking two groups mentioned above into consideration, genetic variation was partitioned as follows: among groups (3.66%), among populations within groups (4.34%), and within populations (92.00%). Notably, the genetic variations within and among populations were similar in the eastern and western groups (**Table 4**).

**Table 4:** Analysis of molecular variation (AMOVA) for natural *Phyllanthus emblica* populations (Pops) in two groups (eastern and western) based on 16 SSR markers.

Types	Source of Variation	Sum of Squares	Variance Component	Percentage of Variation (%)	*P*
Eastern	Among Pops	170.89	0.67	4.30	<0.001
	Within Pops	1994.25	14.99	95.70	<0.001
	Total	2165.14	15.67	100	
Western	Among Pops	321.57	0.79	4.60	<0.001
	Within Pops	3422.40	16.38	95.40	<0.001
	Total	3743.97	17.16	100	
Two groups	Among groups	138.55	0.63	3.66	<0.001
	Among Pops within groups	492.47	0.75	4.34	<0.001
	Within Pops	5416.65	15.84	92.00	<0.001
	Total	6047.66	17.22	100	

At the river valley level, the genetic differentiation (PhiPT) was the largest (0.081) between the Nu and Jinsha Rivers, and the smallest (0.010) between the Longchuan and Lancang River valleys (**Table 5**). At the population level, the PhiPTs between paired populations from the western group as well as from the eastern group were mostly lower than those between any paired populations including one from the western and another from the eastern groups (**Supplementary Table S3**). Furthermore, both the Mantel test and the partial Mantel test (**Table 6**) demonstrated a significant correlation between PhiPT and geographic distance regardless of whether environmental distance was controlled for paired populations *(r* = 0.4326, *p* = 0.0002) or not (*r* = 0.4265, *p* = 0.0002). No significant relationship was observed between PhiPT and environmental distance (*r* = 0.0788, *p* = 0.2081).

**Table 5:** Genetic differentiation values (PhiPT) between five river valleys for *Phyllanthus emblica* based on analysis of molecular variation (AMOVA).

Valleys	Longchuan River	Nu River	Lancang River	Yuan River
Nu River	0.030			
Lancang River	0.010	0.020		
Yuan River	0.026	0.059	0.028	
Jinsha River	0.043	0.081	0.039	0.014

**Table 6:** Results of Mantel and partial Mantel tests for correlations between population genetic differentiation values (PhiPT) and geographical distance (Geo) as well as PhiPT and environmental distance for 18 *Phyllanthus emblica* populations.

Genetic differentiation	Matrix	Controlled	*r*	*p*
PhiPT	Geo		0.4326	0.0002
	Env		0.0788	0.2081
	Geo	Env	0.4265	0.0002
	Env	Geo	-0.0093	0.5225

### 3.4 Geographic and environmental drivers of genetic diversity

**Figure 5:**
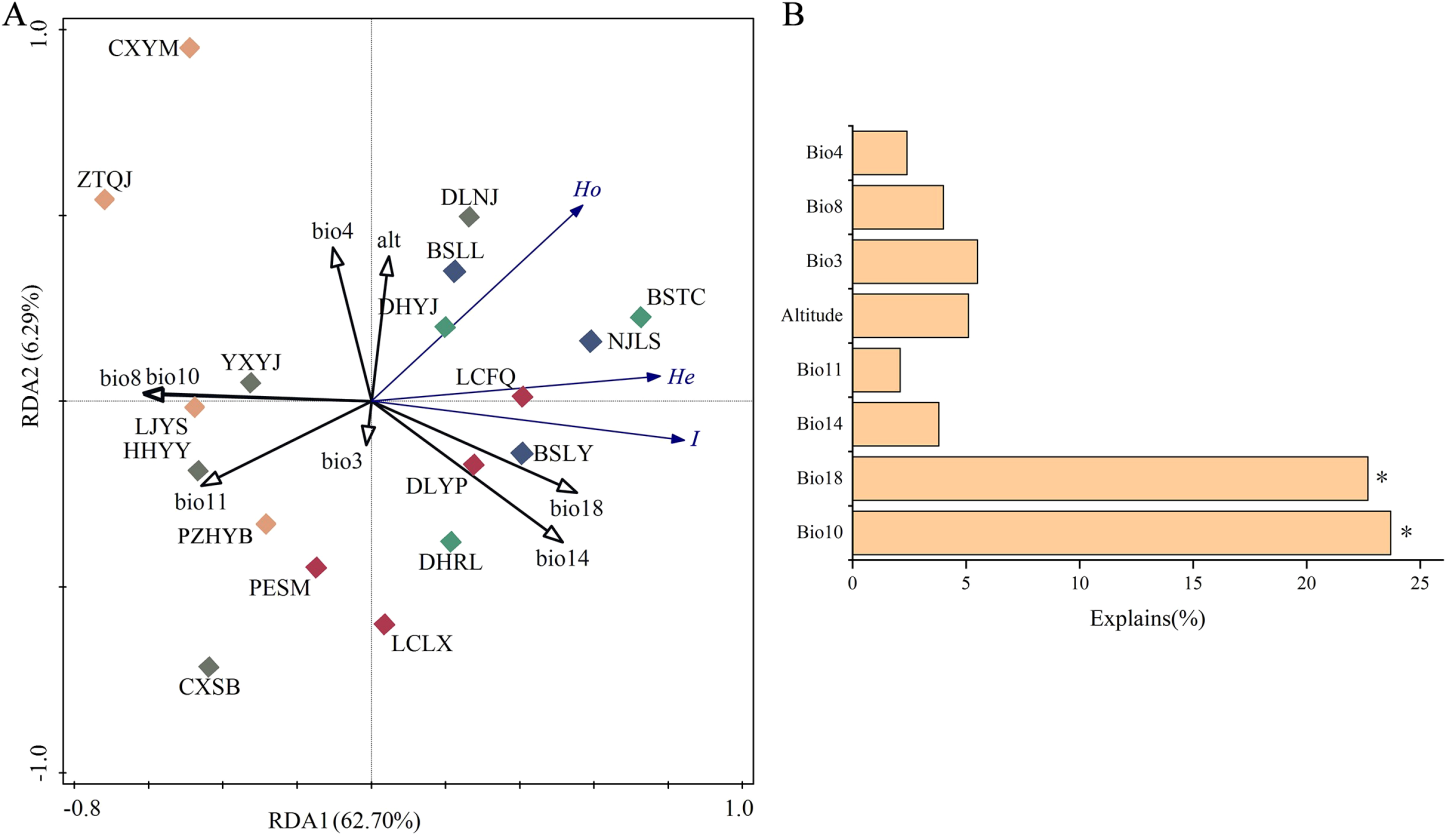
Ordination plots of the redundancy analysis (RDA) for genetic diversity versus environmental factors. **(A)** relationship between genetic diversity and environmental factors; and **(B)** the contribution of environmental factors to genetic diversity variation for *Phyllanthus emblica*. Population information is shown in **Table 1**, and environmental factor information in **Supplementary Table S1**.

Redundancy analysis (RDA) demonstrated that the first and second ordination axes explained 62.70% and 6.29% of the variation in genetic diversity, respectively (**Figure 5A**). *Ho*, *He* and *I* of *P. emblica* populations were in significantly positive correlation with Bio18 (Precipitation of the warmest quarter), and were in negative correlation with Bio10 (Mean temperature of warmest quarter). The Monte Carlo permutation test further showed that only Bio18 and Bio10 had significant effects on genetic diversity (*P*<0.05), with their interpretation degree to the genetic diversity being 23.70% and 22.70%, respectively (**Figure 5B**). Based on *T* test, there existed significant differences in Bio8 and Bio10 between the western and eastern groups (**Table 7**).

**Table 7:** *T* test of differences in 20 environmental factors between the eastern and western groups.

Environment variables	Western	Eastern	*P* value	Environment variables	Western	Eastern	*P* value
Bio1	18.45 ± 0.49	19.95 ± 0.81	0.361	Bio11	12.47 ± 0.56	13.78 ± 0.85	0.380
Bio2	10.97 ± 0.18	10.86 ± 0.27	0.309	Bio12	1346.64 ± 76.96	989.43 ± 92.74	0.990
Bio3	47.58 ± 0.62	46.84 ± 0.89	0.626	Bio13	273.73 ± 21.22	205.00 ± 22.27	0.592
Bio4	430.63 ± 9.53	455.18 ± 14.86	0.930	Bio14	14.00 ± 0.91	10.29 ± 1.36	0.352
Bio5	27.58 ± 0.49	29.79 ± 0.65	0.760	Bio15	83.33 ± 3.08	85.43 ± 4.00	0.659
Bio6	4.52 ± 0.46	6.59 ± 1.09	0.091	Bio16	748.73 ± 53.11	557.43 ± 58.10	0.770
Bio7	23.05 ± 0.21	23.20 ± 0.56	0.052	Bio17	54.27 ± 4.05	40.00 ± 5.79	0.274
Bio8	22.81 ± 0.36	24.65 ± 0.73	0.042	Bio18	748.73 ± 53.11	545.14 ± 64.01	0.954
Bio9	12.91 ± 0.59	14.06 ± 0.93	0.357	Bio19	60.09 ± 7.23	41.86 ± 6.54	0.979
Bio10	22.80 ± 0.36	24.66 ± 0.72	0.045	Altitude	1111.95 ± 71.29	993.71 ± 142.07	0.125

### 3.5 Historical changes of ecological niche for *P. emblica*

**Figure 6:**
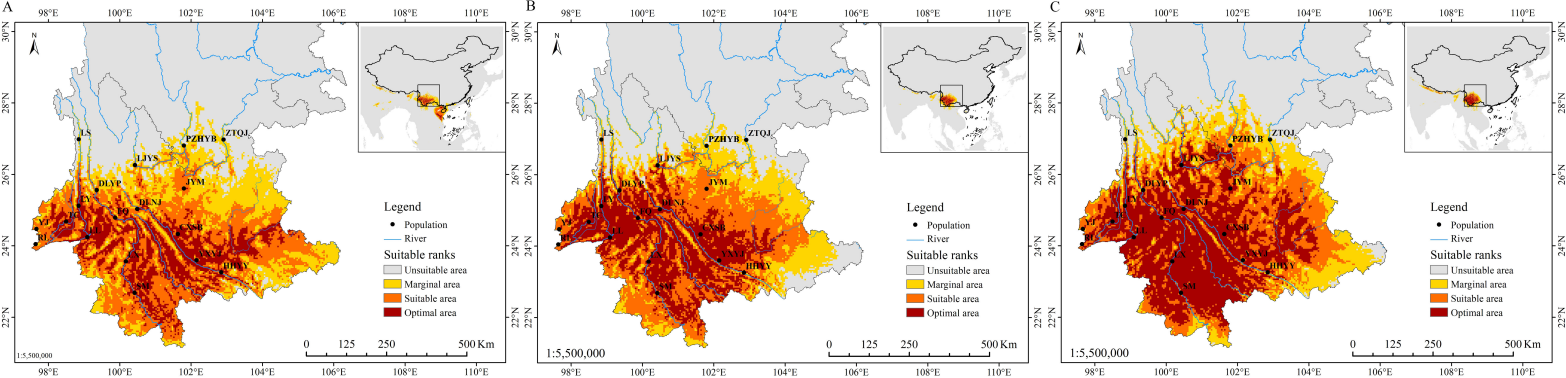
Potential distribution range of *Phyllanthus emblica* in the three periods. **(A)** Last Glacial Maximum; **(B)** Middle Holocene; and **(C)** present. LGM, Last Glacial Maximum; and MH, Middle Holocene.

The mean area under the curve (AUC) value for the current potential distribution zones of *P. emblica* was 0.972 ± 0.001, indicating a better predictive model. From the Last Glacial Maximum (LGM) through the Middle Holocene (MH) to the present, the area of optimal class had shown a trend of increasing (**Figures 6A–C**). The current optimal area was 14.04×10^4^ km^2^ higher than those of LGM and MH, respectively (**Table 8**). The LGM had the smallest area for each potential distribution class. In the past two periods, *P. emblica* was primarily distributed below 26°N in latitude, whereas the contemporary distribution expanded well beyond 26°N, showing a much broader spread across the river valleys studied and their surrounding areas. In particular, it was demonstrated in **Figure 6C** that the optimal distributions on the western side were more continuous than those on the eastern side of the Tanaka-Kaiyong Line, that’s to say, the optimal distribution of *P. emblica* on the eastern side was more fragmented at present.

**Table 8:** Areas of suitable zone of in the three period (×10^4^km^2^).

Period	LGM	MH	Present
Unsuitable area	53.52	53.61	49.40
Marginal area	9.12	7.37	6.90
Suitable area	11.01	10.16	9.69
Optimal area	6.38	8.89	14.04

## 4 Discussion

### 4.1 Genetic diversity

This study revealed high genetic diversity across 18 natural *P. emblica* populations in Yunnan, China, based on 16 SSR loci. The mean number of alleles per locus (*N*a), the observed heterozygosity (*H*o) and the expected heterozygosity (*H*e) were 6.990, 0.790 and 0.666 respectively (**Table 3**). These values exceed those reported for two populations in Thailand (5 SSR loci; *N*a=4.900, *H*o=0.548, *H*e=0.619; Pandey and Changtragoon, 2012), and 20 individuals in India (15 SSR loci; *N*a=4.000, *H*e=0.607; Geethika et al., 2018). However, they were lower than values from 10 natural populations in Yunnan and Guangxi, China (20 EST-SSR loci; *N*a: 10.425, *H*e: 0.792; Liu et al., 2020). Differences in genetic diversity parameters among these studies likely stem not only from variations in the number of populations, areas studied, and SSR markers used, but also from complex ploidy of *P. emblica* (diploid, polyploid, or mixed), a factor known to significantly influence such results (Kosman and Leonard, 2005). Notably, ploidy status was unspecified in the three comparative studies, all of which employed diploid-specific analytical softwares for genetic diversity estimation. This methodological limitation is critical, as standard diploid frameworks cannot accurately resolve heterozygous genotypes in polyploids, potentially biasing diversity estimates (Meirmans et al., 2018). Nevertheless, the *P. emblica* populations in this study exhibit high genetic diversity.

Among the five river valleys, *P. emblica* populations in the Longchuan, Nu and Lancang River valleys exhibited higher level of genetic diversity than those in the Jinsha and Yuan River valleys (**Table 3**). This could be explained by the deduction from **Figure 6** that the Nu and Lancang River valleys may serves as refuge for *P. emblica* during the LGM and MH. No clearly consistent trend was observed in the changes of genetic diversity from upstream to downstream across five river valleys. For example, the Shannon-Wiener diversity index (*I*) exhibited a decline in the Longchuan, Lancang and Yuan River valleys, maintained nearly stable in the Nu River valley, and displayed no discernible pattern in the Jinsha River valley. These differences may be attributed to a complex interplay of altitude (Liang et al., 2008), microclimate (Zhao et al., 2021), microtopography (Ohsawa et al., 2008), and soil properties (Yang et al., 2018), etc. Interestingly, genetic diversity in *P. emblica* was significantly influenced by Bio18 (Precipitation of the warmest quarter) and Bio10 (Mean temperature of the warmest quarter) in this study (**Figure 5**). Furthermore, the absence of a consistent upstream-downstream genetic diversity trend may also stem from pollen- and seed-mediated gene flow shaped by the landscape complexity in the region (Cruzan and Hendrickson, 2020).

### 4.2 Genetic differentiation

The present study revealed that 18 P*. emblica* populations from Yunnan could be divided into two groups: eastern and western, corresponding to the biogeographical partitioning across the Tanaka-Kaiyong Line, a major floristic boundary. The genetic differentiation were significant between the eastern and western groups (**Table 4**, **Supplementary Table S3**). Previous studies of perennial herbs (Zhang et al., 2006), shrubs (Fan et al., 2013), and deciduous trees (Tian et al., 2015) in phylogeography have also demonstrated distinct intraspecific genetic patterns on both sides of the Tanaka-Kaiyong Line. This phenomenon may be attributed to the environmental disparity on eastern and western sides of the Tanaka-Kaiyong Line. The climate is mainly shaped by the East Asian monsoon on the eastern side, while by the Southeast Asian monsoon on the western side (Fan et al., 2013; Tian et al., 2015). Fossil assemblages, palaeogeographic data, and paleoclimate evidence also indicate that Tanaka-Kaiyong Line of biogeographic importance stems from historical tectonic activity and/or prolonged environmental differentiation, effectively restricting plant dispersal and gene flow between the eastern and western populations (Li and Li, 1997; Zhao and Gong, 2015).

In the present study, further explanations can be described in more detail. Temperature is higher and rainfall is lower on the eastern side than on the western side of the Tanaka-Kaiyong Line in the current period (**Table 7**), and mean temperature of the warmest quarter and precipitation of the warmest quarter were the most important factors influencing the genetic diversity of *P. emblica* (**Figure 4**). Such environmental heterogeneity likely imposes divergent selection pressure, and amplifying the genetic differentiation between the eastern and western populations.

Notably, the lowest genetic differentiation occurred between the Longchuan and Lancang River valleys. This pattern may be attributed to the geographical configuration of the DHRL and DHYJ populations in Longchuan River valley and LCLX and PESM populations in Langcang River valley, which inhabit the middle or lower reaches characterized by reduced topographic complexity. Environmental homogeneity across these populations might have reinforced genetic cohesion via stabilizing selection. Furthermore, the absence of significant mountain barriers in these regions likely facilitated sustained gene flow via pollen or seed dispersal. Pollen flow, particularly in wind-pollinated species, typically exhibits long dispersal distances and great permeability compared to seed flow (e.g., gravity/rodent-dispersed plants) (Wang et al., 2012). The effectiveness of both dispersal modes is contingent upon the severity of topographic barriers (Gugger et al., 2008). Additionally, river corridors can facilitate seed dispersal (Nurtaza et al., 2024; Perez-Garcia et al., 2025). Collectively, the open topography and river networks likely explain the low genetic differentiation.

Similarly, relatively low genetic differentiation was observed between the Yuan and Jinsha River valleys. Paleogeological evidence indicates that the Jinsha River was once a tributary of the Yuan River, and was isolated from the Yuan River due to the reorganization of the ancient drainage systems associated with river capture and flow reversal events (Clark et al., 2004). These tectonic processes, which predated or coincided with the Miocene uplift (Clark et al., 2004; Yue et al., 2012), likely preserved ancestral genetic connectivity between *P. emblica* populations in these two valleys, explaining their limited contemporary genetic differentiation.

### 4.3 Distribution dynamics under climate change

Understanding changes in the potential distribution patterns of a species is crucial for assessing the impacts of climate change and developing effective conservation strategies (Deb et al., 2017). Ecological niche analyses showed that the size of the habitable zone of *P. emblica* gradually increased from the Last Glacial Maximum (LGM) to the present (**Table 8**). Studies of Tierney et al (Tierney et al., 2020). and Osman et al. (2021) indicate that the mean temperatures during the LGM and the MH were 6.1°C and 0.5-1.5°C lower than that at the present, respectively. Climate warming has led to an upward shift in the suitable altitude range, significantly expanding the suitable habitats for *P. emblica* in Yunnan. Overall, the species exhibits a low extinction risk in the context of future global warming.

These highly diverse *P. emblica* populations constitute a valuable genetic resource. Under the background of global climate change, exploiting their rich genetic variation can facilitate the selection of climate-resilient, high-quality varieties, and support breeding programs for high-yielding cultivars with specific medicinal values (Swarup et al., 2021). Furthermore, in ecological restoration initiatives, utilizing seeds from these genetically diverse populations ensures rehabilitated stands maintain sufficient genetic variation. This inherent diversity enhances population resilience against future environmental changes, such as climate shifts and emergence of pests or diseases (Kistner et al., 2018).

## 5 Conclusions

High genetic diversity of *P. emblica* was identified across five river valleys in Yunnan, China using SSR markers. The genetic diversity could potentially be influenced by mean temperature of warmest quarter and precipitation of the warmest quarter. Significant genetic differentiation was observed between the western and eastern *P. emblica* populations, potentially due to geographic isolation and environmental heterogeneity, with the Tanaka-Kaiyong Line possibly acting as a contributing barrier. The distribution of *P. emblica* in the eastern region is more fragmented than that in the western region. Given that the natural populations of *P. emblica* have not yet reached an endangered level, it is recommended that *in situ* conservation be the primary conservation approach in the future. Populations with high genetic diversity such as those in the Nu and Longchuan River valleys should be given the highest conservation priority. Concurrently, integrating genetic diversity and genetic structure with fruit morphological and quality characteristics in germplasm collection and evaluation will facilitate the improvement of *P. emblica* germplasms.

## Data Availability

The datasets presented in this study can be found in online repositories. The names of the repository/repositories and accession number(s) can be found below: https://www.ncbi.nlm.nih.gov/genbank/, MK017818 https://www.ncbi.nlm.nih.gov/genbank/, MK017800 https://www.ncbi.nlm.nih.gov/genbank/, MK017799 https://www.ncbi.nlm.nih.gov/genbank/, MK017801 https://www.ncbi.nlm.nih.gov/genbank/, MK017802 https://www.ncbi.nlm.nih.gov/genbank/, MK017803 https://www.ncbi.nlm.nih.gov/genbank/, MK017804 https://www.ncbi.nlm.nih.gov/genbank/, MKO17805 https://www.ncbi.nlm.nih.gov/genbank/, MK017806 https://www.ncbi.nlm.nih.gov/genbank/, MKO17807 https://www.ncbi.nlm.nih.gov/genbank/, MK017808 https://www.ncbi.nlm.nih.gov/genbank/, MK017809 https://www.ncbi.nlm.nih.gov/genbank/, MK017810 https://www.ncbi.nlm.nih.gov/genbank/, MK017811 https://www.ncbi.nlm.nih.gov/genbank/, MK017813 https://www.ncbi.nlm.nih.gov/genbank/, MK017814.

## References

[B1] Badgley C. Smiley T. M. Terry R. Davis E. B. DeSantis L. R. G. Fox D. L.. (2017). Biodiversity and topographic complexity: Modern and geohistorical perspectives. Trends Ecol. Evol. 32, 211–226. doi: 10.1016/j.tree.2016.12.010, PMID: 28196688 PMC5895180

[B2] Bankevich A. Nurk S. Antipov D. Gurevich A. A. Dvorkin M. Kulikov A. S.. (2012). SPAdes: A new genome assembly algorithm and its applications to single-cell sequencing. J. Comput. Biol. 19, 455–477. doi: 10.1089/cmb.2012.0021, PMID: 22506599 PMC3342519

[B3] Chaphalkar R. Apte K. G. Talekar Y. Ojha S. K. Nandave M. (2017). Antioxidants of *Phyllanthus emblica* L. bark extract provide hepatoprotection against ethanol-induced hepatic damage: A comparison with silymarin. Oxid. Med. Cell. Longev. 1, 3876040. doi: 10.1155/2017/3876040, PMID: 28168009 PMC5267079

[B4] Clark M. K. Schoenbohm L. M. Royden L. H. Whipple K. X. Burchfiel B. C. Zhang X.. (2004). Surface uplift, tectonics, and erosion of eastern Tibet from largescale drainage patterns. Tectonics 23, 2002TC001402. doi: 10.1029/2002TC001402

[B5] Cruzan M. B. Hendrickson E. C. (2020). Landscape genetics of plants: Challenges and opportunities. Plant Commun. 1, 100100. doi: 10.1016/j.xplc.2020.100100, PMID: 33367263 PMC7748010

[B6] Deb J. C. Phinn S. Butt N. McAlpine C. A. (2017). The impact of climate change on the distribution of two threatened Dipterocarp trees. Ecol. Evol. 7, 2238–2248. doi: 10.1002/ece3.2846, PMID: 28405287 PMC5383467

[B7] Earl D. A. vonHoldt B. M. (2012). STRUCTURE HARVESTER: A website and program for visualizing STRUCTURE output and implementing the Evanno method. Conserv. Genet. Resour. 4, 359–361. doi: 10.1007/s12686-011-9548-7

[B8] Fan D. M. Yue J. P. Nie Z. L. Li Z. M. Comes H. P. Sun H. (2013). Phylogeography of *Sophora davidii* (Leguminosae) across the ‘Tanaka-Kaiyong Line’, an important phytogeographic boundary in Southwest China. Mol. Ecol. 22, 4270–4288. doi: 10.1111/mec.12388, PMID: 23927411

[B9] Favre A. Päckert M. Pauls S. U. Jähnig S. C. Uhl D. Michalak I. (2015). The role of the uplift of the Qinghai-Tibetan Plateau for the evolution of Tibetan biotas. Biol. Rev. 90, 236–253. doi: 10.1111/brv.12107, PMID: 24784793

[B10] Gao X. X. Liu J. Huang Z. H. (2022). The impact of climate change on the distribution of rare and endangered tree *Firmiana kwangsiensis* using the Maxent modeling. Ecol. Evol. 12, e9165. doi: 10.1002/ece3.9165, PMID: 35919389 PMC9336174

[B11] Geethika E. Triveni H. N. Srirama R. Siva R. Siddappa S. Ravikanth G. (2018). Development and characterization of microsatellite markers for *Phyllanthus emblica* Linn, important nontimber forest product species. J. Genet. 97, 1001–1006. doi: 10.1007/s12041-018-0979-8, PMID: 30262713

[B12] Gugger P. F. McLachlan J. S. Manos P. S. Clark J. S. (2008). Inferring long-distance dispersal and topographic barriers during post-glacial colonization from the genetic structure of red maple (*Acer rubrum* L.) in New England. J. Biogeogr. 35, 1665–1673. doi: 10.1111/j.1365-2699.2008.01915.x

[B13] Guisan A. Zimmermann N. E. (2000). Predictive habitat distribution models in ecology. Ecol. Model. 135, 147–186. doi: 10.1016/S0304-3800(00)00354-9

[B14] Guo J. J. Shang S. B. Wang C. S. Zhao Z. G. Zeng J. (2017). Twenty microsatellite markers for the endangered *Vatica mangachapoi* (Dipterocarpaceae). Appl. Plant Sci. 5, 1600134. doi: 10.3732/apps.1600134, PMID: 28224060 PMC5315383

[B15] He K. Jiang X. (2014). Sky islands of southwest China. I: an overview of phylogeographic patterns. Chin. Sci. Bull. 59, 1–13. doi: 10.1007/s11434-013-0089-1

[B16] Hewitt G. M. (2000). The genetic legacy of the quaternary ice ages. Nature 405, 907–913. doi: 10.1038/35016000, PMID: 10879524

[B17] Hulce D. Li X. Snyder-Leiby T. Liu C. J. (2011). GeneMarker^®^ genotyping software: Tools to increase the statistical power of DNA fragment analysis. J. Biomole. Tech. 22, S35.

[B18] Jia J. Zeng L. Gong X. (2016). High genetic diversity and population differentiation in the critically endangered plant species *Trailliaedoxa gracilis* (Rubiaceae). Plant Mol. Biol. Rep. 34, 327–338. doi: 10.1007/s11105-015-0924-4

[B19] Jinga P. Ashley M. V. (2018). A mountain range is a strong genetic barrier between populations of *Afzelia quanzensis* (pod mahogany) with low genetic diversity. Tree Genet. Genomes 14, 1–10. doi: 10.1007/s11295-017-1217-x

[B20] Kamvar Z. N. Tabima J. F. Grünwald N. J. (2014). Poppr: An R package for genetic analysis of populations with clonal, partially clonal, and/or sexual reproduction. Peer J. 2, e281. doi: 10.7717/peerj.281, PMID: 24688859 PMC3961149

[B21] Kistner E. Kellner O. Andresen J. Todey D. Morton L. W. (2018). Vulnerability of specialty crops to short-term climatic variability and adaptation strategies in the Midwestern USA. Climatic Change 146, 145–158. doi: 10.1007/s10584-017-2066-1

[B22] Kosman E. Leonard K. J. (2005). Similarity coefficients for molecular markers in studies of genetic relationships between individuals for haploid, diploid, and polyploid species. Mol. Ecol. 14, 415–424. doi: 10.1111/j.1365-294X.2005.02416.x, PMID: 15660934

[B23] Krishnaiah D. Devi T. Bono A. (2009). Studies on phytochemical constituents of six Malaysian medicinal plants. J. Med. Plants Res. 3, 67–72.

[B24] Kumar S. Stecher G. Tamura K. (2016). MEGA7: molecular evolutionary genetics analysis version 7.0 for bigger datasets. Mol. Biol. Evol. 33, 1870–1874. doi: 10.1093/molbev/msw054, PMID: 27004904 PMC8210823

[B25] Labbé J. Murat C. Morin E. Le Tacon F. Martin F. (2011). Survey and analysis of simple sequence repeats in the *Laccaria bicolor* genome, with development of microsatellite markers. Curr. Genet. 57, 75–88. doi: 10.1007/s00294-010-0328-9, PMID: 21132299

[B26] Li X. W. Li J. (1997). The Tanaka-Kaiyong line - An important floristic line for the study of the flora of East Asia. Ann. Mo. Bot. Gard. 84, 888–892. doi: 10.2307/2992033

[B27] Li Y. Zhang X. Fang Y. (2019). Landscape features and climatic forces shape the genetic structure and evolutionary history of an oak species (*Quercus chenii*) in East China. Front. Plant Sci. 10. doi: 10.3389/fpls.2019.01060, PMID: 31552065 PMC6734190

[B28] Li Q. M. Zhao J. L. (2007). Genetic diversity of *Phyllanthus emblica* populations in dry-hot valleys in Yunnan. Bio. Sci. 15, 84–91. doi: 10.1360/biodiv

[B29] Liang Y. Liu J. Zhang S. P. Wang S. J. Guo W. H. Wang R. Q. (2008). Genetic diversity of the invasive plant *Coreopsis grandiflora* at different altitudes in Laoshan Mountain, China. Can. J. Plant Sci. 88, 831–837. doi: 10.4141/CJPS07020

[B30] Liu X. Ma Y. Wan Y. Li Z. Ma H. (2020). Genetic diversity of *Phyllanthus emblica* from two different climate type areas. Front. Plant Sci. 11. doi: 10.3389/fpls.2020.580812, PMID: 33329643 PMC7734338

[B31] López-González N. Bobo-Pinilla J. Padilla-García N. Loureiro J. Castro S. Rojas-Andrés B. M.. (2021). Genetic similarities versus morphological resemblance: Unraveling a polyploid complex in a Mediterranean biodiversity hotspot. Mol. Phylogenet. Evol. 155, 107006. doi: 10.1016/j.ympev.2020.107006, PMID: 33160038

[B32] Ma Q. G. Wang L. Liu R. H. Yuan J. B. Xiao H. Shen Z. Y.. (2024). *Phyllanthus emblica* Linn: A comprehensive review of botany, traditional uses, phytonutrients, health benefits, quality markers, and applications. Food Chem. 446, 138891. doi: 10.1016/j.foodchem.2024.138891, PMID: 38432135

[B33] Markwith S. H. Stewart D. J. Dyer J. L. (2006). TETRASAT: a program for the population analysis of allotetraploid microsatellite data. Mol. Ecol. Notes 6, 586–589. doi: 10.1111/j.1471-8286.2006.01345.x

[B34] Meirmans P. G. Liu S. van Tienderen P. H. (2018). The analysis of polypoid genetic data. J. Hered. 109, 283–296. doi: 10.1093/jhered/esy006, PMID: 29385510

[B35] Meng H. H. Su T. Gao X. Y. Li J. Jiang X. L. Sun H.. (2017). Warm–cold colonization: response of oaks to uplift of the Himalaya–Hengduan Mountains. Mol. Ecol. 26, 3276–3294. doi: 10.1111/mec.14092, PMID: 28281334

[B36] Morris C. (2015). Multivariate analysis of ecological data using Canoco 5, 2nd Edition. Afr. J. Range For. Sci. 32, 289–290. doi: 10.2989/10220119.2015.1015053

[B37] Nakanishi A. Takeuchi T. Ueno S. Nishimura N. Tomaru N. (2020). Spatial variation in bird pollination and its mitigating effects on the genetic diversity of pollen pools accepted by *Camellia japonica* trees within a population at a landscape level. Heredity 124, 170–181. doi: 10.1038/s41437-019-0262-7, PMID: 31485029 PMC6906363

[B38] Nurtaza A. Dyussembekova D. Shevtsov A. Islamova S. Samatova I. Koblanova S.. (2024). Assessing genetic variability and population structure of *Alnus glutinosa* (black alder) in Kazakhstan using SSR markers. Plants 13, 3032. doi: 10.3390/plants13213032, PMID: 39519950 PMC11548218

[B39] Ohsawa T. Saito Y. Sawada H. Ide Y. (2008). Impact of altitude and topography on the genetic diversity of *Quercus serrata* populations in the Chichibu Mountains, central Japan. Flora 203, 187–196. doi: 10.1016/j.flora.2007.02.007

[B40] Osman M. B. Tierney J. E. Zhu J. Tardif R. Hakim G. J. (2021). Globally resolved surface temperatures since the Last Glacial Maximum. Nature 599, 239–244. doi: 10.1038/s41586-021-03984-4, PMID: 34759364

[B41] Pandey M. Changtragoon S. (2012). Isolation and characterization of microsatellites in a medicinal plant, *Phyllanthus emblica* (Euphorbiaceae). Am. J. Bot. 99, e468–e469. doi: 10.3732/ajb.1200157, PMID: 23196395

[B42] Peakall R. Smouse P. E. (2006). GENALEX 6: Genetic analysis in Excel. Population genetic software for teaching and research. Mol. Ecol. Notes 6, 288–295. doi: 10.1111/j.1471-8286.2005.01155.x, PMID: 22820204 PMC3463245

[B43] Perez-Garcia L. Pérez-Alquicira J. Rico Y. Vargas-Ponce O. Montti L. Ruiz-Sanchez E. (2025). Despite forest fragmentation, river connectivity maintains gene flow and diversity in *Guadua trinii*, a woody bamboo of the Atlantic Forest in Argentina. Hydrobiologia 852, 1637–1650. doi: 10.1007/s10750-024-05764-3

[B44] Pritchard J. K. Stephens M. Donnelly P. (2000). Inference of population structure using multilocus genotype data. Genetics 155, 945–959. doi: 10.1093/genetics/155.2.945, PMID: 10835412 PMC1461096

[B45] Rahman M. S. Sultana S. S. Hassan M. A. (2021). Variable chromosome number and ploidy level of five *Phyllanthus* species in Bangladesh. Cytologia 86, 143–148. doi: 10.1508/cytologia.86.143

[B46] Saini R. Sharma N. Oladeji O. S. Sourirajan A. Dev K. Zengin G.. (2022). Traditional uses, bioactive composition, pharmacology, and toxicology of *Phyllanthus emblica* fruits: A comprehensive review. J. Ethnopharmacol. 282, 114570. doi: 10.1016/j.jep.2021.114570, PMID: 34480995

[B47] Swarup S. Cargill E. J. Crosby K. Flagel L. Kniskern J. Glenn K. (2021). Genetic diversity is indispensable for plant breeding to improve crops. Crop Sci. 61, 839–852. doi: 10.1002/csc2.20377

[B48] Tang R. Liu E. Zhang Y. Schinnerl J. Sun W. Chen G. (2020). Genetic diversity and population structure of *Amorphophallus albus*, a plant species with extremely small populations (PSESP) endemic to dry-hot valley of Jinsha River. BMC Genet. 21, 102. doi: 10.1186/s12863-020-00910-x, PMID: 32919456 PMC7488774

[B49] Thapa R. Bayer R. J. Mandel J. R. (2021). Genetic diversity, population structure, and ancestry estimation in the *Antennaria rosea* (Asteraceae: Gnaphalieae) polyploid agamic complex. Taxon 70, 139–152. doi: 10.1002/tax.12420

[B50] Tian B. Zhou Z. Du F. K. He C. Xin P. Ma H. (2015). The Tanaka Line shaped the phylogeographic pattern of the cotton tree (*Bombax ceiba*) in southwest China. Biochem. Syst. Ecol. 60, 150–157. doi: 10.1016/j.bse.2015.04.014

[B51] Tierney J. E. Zhu J. King J. Malevich S. B. Hakim G. J. Poulsen C. J. (2020). Glacial cooling and climate sensitivity revisited. Nature 584, 569–573. doi: 10.1038/s41586-020-2617-x, PMID: 32848226

[B52] Untergasser A. Cutcutache I. Koressaar T. Ye J. Faircloth B. C. Remm M.. (2012). Primer 3–New capabilities and interfaces. Nucleic Acids Res. 40, e115. doi: 10.1093/nar/gks596, PMID: 22730293 PMC3424584

[B53] Van-Puyvelde K. Van-Geert A. Triest L. (2010). ATETRA, a new software program to analyse tetraploid microsatellite data: Comparison with TETRA and TETRASAT. Mol. Ecol. Resour. 10, 331–334. doi: 10.1111/j.1755-0998.2009.02748.x, PMID: 21565028

[B54] Variya B. C. Bakrania A. K. Patel S. S. (2016). *Emblica officinalis* (Amla): A review for its phytochemistry, ethnomedicinal uses and medicinal potentials with respect to molecular mechanisms. Pharmacol. Res. 111, 180–200. doi: 10.1016/j.phrs.2016.06.013, PMID: 27320046

[B55] Wang R. Compton S. G. Shi Y. S. Chen X. Y. (2012). Fragmentation reduces regional-scale spatial genetic structure in a wind-pollinated tree because genetic barriers are removed. Ecol. Evol. 2, 2250–2261. doi: 10.1002/ece3.344, PMID: 23139883 PMC3488675

[B56] Wang Y. X. Ding K. Zhou R. L. (2017). Spatial estimation of the terrestrial biodiversity enrichment based on topographical and water–energy indexes: As Yunnan for example. J. Yunnan Uni. 39, 481–493. doi: 10.1016/j.phrs.2016.06.013, PMID: 27320046

[B57] Wu M. Cheng Y. Jiang C. X. Zhang M. S. Shi T. Zhao C. (2024). Phylogeography of *Morella nana*: The Wumeng Mountains as a natural geographical isolation boundary on the Yunnan-Guizhou Plateau. Ecol. Evol. 14, e11566. doi: 10.1002/ece3.11566, PMID: 38983704 PMC11232048

[B58] Xing Y. Ree R. H. (2017). Uplift-driven diversification in the Hengduan Mountains, a temperate biodiversity hotspot. Proc. Natl. Acad. Sci U.S.A. 114, 3444–3451. doi: 10.1073/pnas.1616063114, PMID: 28373546 PMC5410793

[B59] Yang J. Vázquez L. Feng L. Liu Z. Zhao G. (2018). Climatic and soil factors shape the demographical history and genetic diversity of a deciduous oak (*Quercus liaotungensis*) in Northern China. Front. Plant Sci. 9. doi: 10.3389/fpls.2018.01534, PMID: 30410498 PMC6209687

[B60] Yin R. P. Huang J. C. Yin G. S. Wu J. H. (2018). Phenotypic diversity of fruits, seeds and offspring seedlings in natural populations of *Phyllanthus emblica* from Western Yunnan. J. Yunnan Uni. 40, 174–182. doi: 10.7540/j.ynu.20170255

[B61] Yue L. L. Chen G. Sun W. B. Sun H. (2012). Phylogeography of *Buddleja crispa* (Buddlejaceae) and its correlation with drainage system evolution in southwestern China. Am. J. Bot. 99, 1726–1735. doi: 10.3732/ajb.1100506, PMID: 23024123

[B62] Zeng J. Zou Y. P. Bai J. Y. Zheng H. S. (2002). Preparation of total DNA from ‘recalcitrant plant taxa’. Acta Bot. Sin. 44, 694–697.

[B63] Zhang L. Li Q. J. Li H. T. Chen J. Li D. Z. (2006). Genetic diversity and geographic differentiation in *Tacca chantrieri* (Taccaceae): an autonomous selfing plant with showy floral display. Ann. Bot. 98, 449–457. doi: 10.1093/aob/mcl123, PMID: 16790462 PMC2803468

[B64] Zhao Y. J. Gong X. (2015). Genetic divergence and phylogeographic history of two closely related species (*Leucomeris decora* and *Nouelia insignis*) across the ‘Tanaka Line’ in Southwest China. BMC Evol. Biol. 15, 134. doi: 10.1186/s12862-015-0374-5, PMID: 26153437 PMC4495643

[B65] Zhao W. Wang X. Li L. Li J. Yin H. Zhao Y.. (2021). Evaluation of environmental factors affecting the genetic diversity, genetic structure, and the potential distribution of *Rhododendron aureum* Georgi under changing climate. Ecol. Evol. 11, 12294–12306. doi: 10.1002/ece3.7803, PMID: 34594500 PMC8462154

[B66] Zhu X. Y. Jiang X. Chen Y. Li C. C. Ding S. Zhang X. J.. (2025). Prediction of potential distribution and response of *Changium smyrnioides* to climate change based on optimized MaxEnt model. Plants 14, 743. doi: 10.3390/plants14050743, PMID: 40094718 PMC11901656

