# Supplementary material for: Geographic isolation and environmental heterogeneity shape population genetic differentiation of a medicinal plant, amla (Phyllanthus emblica L.) in river valleys of Yunnan, China
Source: Front Plant Sci. 2025 Sep 10;16:1648822. doi: 10.3389/fpls.2025.1648822 (PMC12457440; doi:10.3389/fpls.2025.1648822)
Supplement: Supplementary file 1 [file Table1.docx]

**Supplementary materials**

Table S1 Environmental variables used in the present study.

| Environment variables | Description | Unit | Environment variables | Description | Unit |
| --- | --- | --- | --- | --- | --- |
| Bio 1 | Annual mean temperature | ℃ | **Bio 11** | **Mean temperature of the coldest quarter** | ℃ |
| Bio 2 | Mean diurnal range | ℃ | Bio 12 | Annual precipitation | mm |
| **Bio 3** | **Isothermality (Bio 2/Bio 7)×100** | % | Bio 13 | Precipitation of wettest month | mm |
| **Bio 4** | **Temperature seasonality** | ℃ | **Bio 14** | **Precipitation of driest month** | mm |
| Bio 5 | Max temperature of the warmest month | ℃ | Bio 15 | Precipitation seasonality | % |
| Bio 6 | Minimum temperature of the coldest month | ℃ | Bio 16 | Precipitation of the wettest quarter | mm |
| Bio 7 | Temperature annual range | ℃ | Bio 17 | Precipitation of the driest quarter | mm |
| **Bio 8** | **Mean temperature of the wettest quarter** | ℃ | **Bio 18** | **Precipitation of the warmest quarter** | mm |
| Bio 9 | Mean temperature of the driest quarter | ℃ | Bio 19 | Precipitation of the coldest quarter | mm |
| **Bio 10** | **Mean temperature of the warmest quarter** | ℃ | **Alt** | **Altitude** | m |

Table S2

Analysis of molecular variation (AMOVA) for *Phyllanthus emblica* populations in five river valleys based on 16 SSR markers.

| Types | Source of Variation | Sum of Squares | Variance Component | Percentage of Variation (%) | *P* |
| --- | --- | --- | --- | --- | --- |
| Whole | Among Pops | 631.01 | 1.06 | 6.30 | <0.001 |
|  | Within Pops | 5416.65 | 15.84 | 93.70 | <0.001 |
|  | Total | 6047.66 | 16.90 | 100 |  |
| Longchuan River | Among Pops | 73.00 | 1.00 | 5.69 | <0.001 |
|  | Within Pops | 942.80 | 16.54 | 94.31 | <0.001 |
|  | Total | 1015.80 | 17.54 | 100 |  |
| Nu River | Among Pops | 51.93 | 0.46 | 2.65 | <0.001 |
|  | Within Pops | 957.80 | 16.80 | 97.35 | <0.001 |
|  | Total | 1009.73 | 17.26 | 100 |  |
| Lancang River | Among Pops | 94.56 | 0.77 | 4.59 | <0.001 |
|  | Within Pops | 1220.70 | 16.06 | 95.41 | <0.001 |
|  | Total | 1315.26 | 16.83 | 100 |  |
| Yuan River | Among Pops | 95.60 | 0.82 | 4.99 | <0.001 |
|  | Within Pops | 1180.75 | 15.54 | 95.01 | <0.001 |
|  | Total | 1276.35 | 16.35 | 100 |  |
| Jinsha River | Among Pops | 81.41 | 0.62 | 4.08 | <0.001 |
|  | Within Pops | 1114.60 | 14.67 | 95.92 | <0.001 |
|  | Total | 1196.01 | 15.29 | 100 |  |
| Five valleys | Among valleys | 234.50 | 0.39 | 2.32 | <0.001 |
|  | Among Pops within valleys | 396.51 | 0.73 | 4.32 | <0.001 |
|  | Within Pops | 5416.65 | 15.84 | 93.36 | <0.001 |
|  | Total | 6047.66 | 16.96 | 100 |  |

Table S3 Genetic differentiation values (PhiPT) of paired populations of *Phyllanthus emblica* infive river valleys.

| **Valleys**  **Population** | **Longchuan River** | | |  | | **Nu River** | | |  | | **Lancang River** | | | |  | | **Yuan River** | | | |  | | **Jiansha River** | | | |
| --- | --- | --- | --- | --- | --- | --- | --- | --- | --- | --- | --- | --- | --- | --- | --- | --- | --- | --- | --- | --- | --- | --- | --- | --- | --- | --- |
|  | **BSTC** | **DHYJ** | **DHRL** | | **NJLS** | | **BSLY** | **BSLL** | | **DLYP** | | **LCFQ** | **LCLX** | **PESM** | | **DLNJ** | | **CXSB** | **YXYJ** | **HHYY** | | **LJYS** | | **PZHYB** | **CXYM** | **ZTQJ** |
| **BSTC** | - |  |  | |  | |  |  | |  | |  |  |  | |  | |  |  |  | |  | |  |  |  |
| **DHYJ** | 0.068 | - |  | |  | |  |  | |  | |  |  |  | |  | |  |  |  | |  | |  |  |  |
| **DHRL** | 0.036 | 0.065 | - | |  | |  |  | |  | |  |  |  | |  | |  |  |  | |  | |  |  |  |
| **NJLS** | 0.048 | 0.071 | 0.089 | | - | |  |  | |  | |  |  |  | |  | |  |  |  | |  | |  |  |  |
| **BSLY** | 0.030 | 0.067 | 0.059 | | 0.007 | | - |  | |  | |  |  |  | |  | |  |  |  | |  | |  |  |  |
| **BSLL** | 0.028 | 0.076 | 0.056 | | 0.049 | | 0.021 | - | |  | |  |  |  | |  | |  |  |  | |  | |  |  |  |
| **DLYP** | 0.033 | 0.079 | 0.066 | | 0.032 | | 0.010 | 0.045 | | - | |  |  |  | |  | |  |  |  | |  | |  |  |  |
| **LCFQ** | 0.021 | 0.072 | 0.038 | | 0.049 | | 0.014 | 0.009 | | 0.035 | | - |  |  | |  | |  |  |  | |  | |  |  |  |
| **LCLX** | 0.046 | 0.057 | 0.039 | | 0.081 | | 0.069 | 0.053 | | 0.079 | | 0.039 | - |  | |  | |  |  |  | |  | |  |  |  |
| **PESM** | 0.039 | 0.043 | 0.020 | | 0.074 | | 0.061 | 0.058 | | 0.071 | | 0.041 | 0.006 | - | |  | |  |  |  | |  | |  |  |  |
| **DLNJ** | 0.026 | 0.053 | 0.034 | | 0.048 | | 0.038 | 0.009 | | 0.051 | | 0.018 | 0.048 | 0.041 | | - | |  |  |  | |  | |  |  |  |
| **CXSB** | 0.077 | 0.102 | 0.058 | | 0.127 | | 0.101 | 0.077 | | 0.102 | | 0.064 | 0.059 | 0.054 | | 0.066 | | - |  |  | |  | |  |  |  |
| **YXYJ** | 0.064 | 0.063 | 0.045 | | 0.095 | | 0.082 | 0.087 | | 0.095 | | 0.060 | 0.055 | 0.034 | | 0.048 | | 0.047 | - |  | |  | |  |  |  |
| **HHYY** | 0.087 | 0.088 | 0.062 | | 0.126 | | 0.111 | 0.117 | | 0.132 | | 0.089 | 0.069 | 0.044 | | 0.066 | | 0.042 | 0.025 | - | |  | |  |  |  |
| **LJYS** | 0.086 | 0.117 | 0.070 | | 0.145 | | 0.115 | 0.113 | | 0.110 | | 0.089 | 0.080 | 0.058 | | 0.072 | | 0.070 | 0.052 | 0.094 | | - | |  |  |  |
| **PZHYB** | 0.066 | 0.071 | 0.040 | | 0.099 | | 0.078 | 0.068 | | 0.081 | | 0.067 | 0.053 | 0.026 | | 0.048 | | 0.025 | 0.035 | 0.036 | | 0.054 | | - |  |  |
| **CXYM** | 0.087 | 0.114 | 0.047 | | 0.115 | | 0.107 | 0.097 | | 0.105 | | 0.073 | 0.078 | 0.049 | | 0.057 | | 0.049 | 0.006 | 0.052 | | 0.045 | | 0.040 | - |  |
| **ZTQJ** | 0.088 | 0.081 | 0.049 | | 0.116 | | 0.096 | 0.090 | | 0.083 | | 0.070 | 0.068 | 0.050 | | 0.060 | | 0.048 | 0.023 | 0.054 | | 0.048 | | 0.033 | 0.024 | - |

Notes: Population information are shown in Table 1.
